# Prosthodontic Rehabilitation Alternative of Patients with Cleft Lip and Palate (CLP): Two Cases Report

**DOI:** 10.1155/2009/515790

**Published:** 2010-02-11

**Authors:** Emrah Ayna, Emine Göncü Başaran, Köksal Beydemir

**Affiliations:** Department of Prosthodontics, Dental Faculty, Dicle University, Diyarbakır 21280, Turkey

## Abstract

Although patients with cleft lip and palate (CLP) are not seen regularly in general dental practice, this is a frequent congenital anomaly; approximately one in every 800 live births results in a CLP. The cause of CLP is unknown, but possible causes are malnutrition and irradiation during pregnancy, psychological stress, teratogenic agents, infectious agents (viruses), and inheritance. Most clefts are likely caused by multiple genetic and nongenetic factors. Prosthetic reconstruction of the anterior maxilla is important for these patients. This paper describes the prosthetic rehabilitation of two patients with CLP, 19-year-old and 21-year-old women, both with surgically treated CLP. In both, an examination revealed a residual palatal defect of 2 × 3 mm and missing maxillary lateral incisors. The 19-year-old was treated with a fiber-reinforced composite resin-bonded fixed partial denture. The 21-year-old was treated with a removable partial denture with an extracoronal attachment system. The prosthetic rehabilitation of the two patients with CLP was evaluated clinically. In both, well-planned prosthetic, periodontal, and surgical therapy resulted in satisfactory function and esthetics, alleviating their deformities. With education and appropriate recall, the patients should be able to maintain their oral health.

## 1. Introduction

Providing maxillofacial prosthetic treatment for patients with congenital and craniofacial defects not only should address physical and functional deficiencies but also should ideally evaluate the possible psychological effects of these deformities [1].

Over the years, we have observed that patients with partial anodontia, cleft lip and palate, amelogenesis imperfecta, dentinogenesis imperfecta, ectodermal dysplasia, and neurological defects frequently have physical anomalies. These anomalies include, but are not limited to, decreased vertical dimensions of occlusion, decreased facial support, temporomandibular joint symptoms, lack of functional occlusion, altered speech, poor esthetics, teeth sensitivity due to abnormal wear and abrasion, lack of a normal smile line, and altered anatomy in the lower third of the face. These patients often require a combination of dental and medical specialists to improve these functional and esthetic problems. Maxillofacial prosthodontic treatment offers improvement in the appearance, function, and health of patients with congenital and craniofacial defects [1]. 

Although patients with cleft palate may not be seen regularly in general dental practice, this is a frequent congenital anomaly; approximately one in every 800 live births results in a cleft lip and palate [1–4]. The cause of cleft lip and palate is unknown, but possible causes are malnutrition and irradiation during pregnancy, psychological stress, teratogenic agents, infectious agents (viruses), and inheritance [4]. Most clefts are likely caused by multiple genetic and nongenetic factors [5]. Currently, owing to the increased knowledge of craniofacial growth and development and improved surgical and orthodontic treatment, patients with cleft palate receive better care and in a timelier fashion [6]. Therefore, they require less prosthetic intervention. Still, prosthetic treatment retains an important, if somewhat diminished, place in cleft palate care [7].

Congenitally missing anterior teeth are common in cleft palate patients. In unilateral or bilateral clefts, the lateral incisors are the most frequently missing teeth, although the canines and central incisors may also be affected [5]. When present, these teeth may be malformed and malposed. The bone support of teeth adjacent to the cleft is generally compromised [8].

A conventional fixed dental prosthesis can be used in the prosthetic treatment of a unilateral cleft and palate (UCLP) patient. This requires preparing at least one tooth on each side of the edentulous space and placing complete or partial metal-ceramic restorations [9]. Consequently, good function and esthetics can be achieved, and the long-term success is more predictable [5, 7]. However, a removable partial denture with/without extra or intracoronal attachment can also be used in prosthetic treatment, if lip support is increased due to poor bone quality [10].

This clinical report describes two alternative prosthetic treatments for two UCLP patients.

## 2. Case Reports

We treated a 19-year-old woman and a 21-year-old woman with surgically treated UCLP in the Department of Prosthodontics, Dicle University. An examination revealed a residual palatal defect of 2 × 3 mm and missing maxillary lateral incisors in both. The 19-year-old woman was treated with a fiber-reinforced composite resin-bonded fixed partial denture (RBFPD). The 21-year-old woman was treated with a removable partial denture (RPD) with an extracoronal attachment system.

### 2.1. Clinical Procedures Involved in the RPD with Extracoronal Attachment

**Figure 1:**
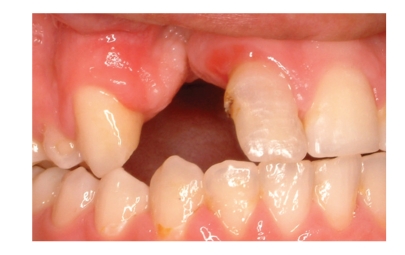
Case 1 with cleft lip and palate.

**Figure 2:**
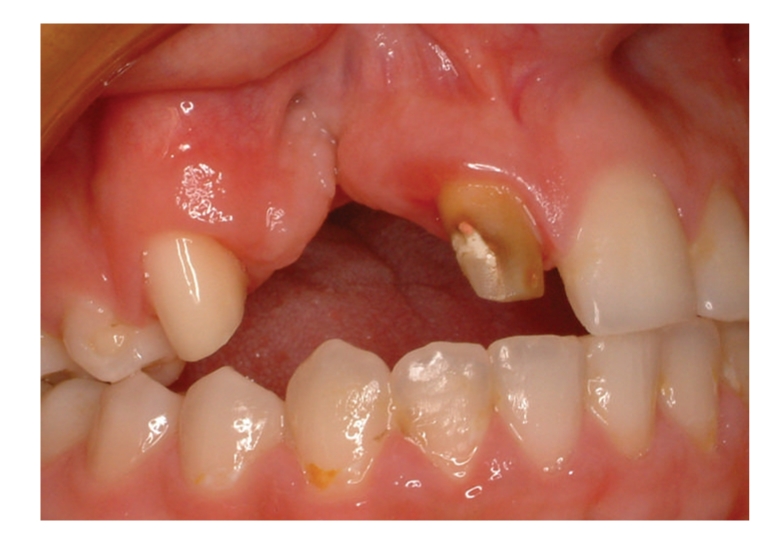
Prepared teeth.

**Figure 3:**
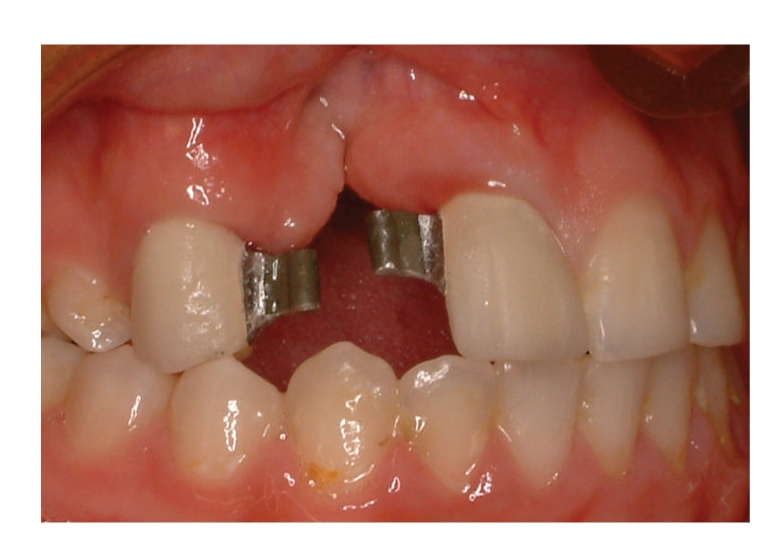
Metal-ceramic crowns with extracoronal attachments.

**Figure 4:**
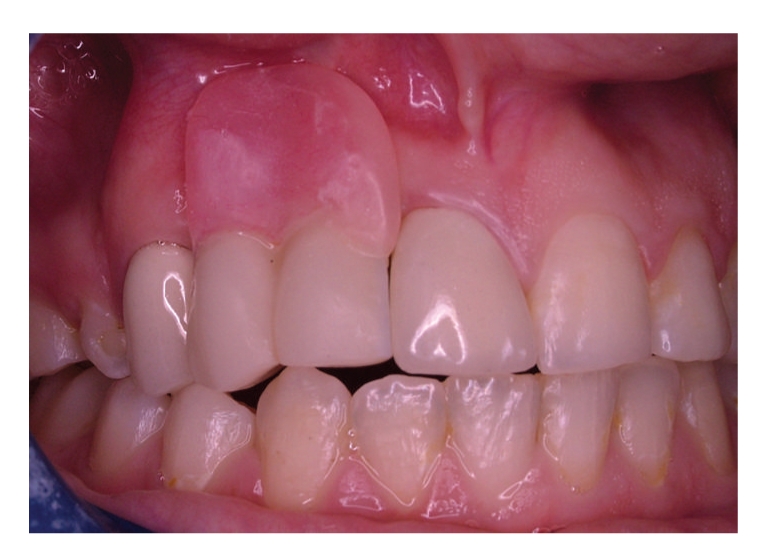
Final restoration.

The radiographic and clinical analyses showed no bone loss around the abutment teeth (Figure 1). The right central incisor and right canine were prepared to receive a unit crown (Figure 2). After routine impression and laboratory procedures, metal-ceramic crowns with extracoronal attachment were luted with polycarboxylate cement (Figure 3). Then, impressions were made for a removable partial denture. The final restorations met both esthetic and functional needs (Figure 4).

### 2.2. Clinical Procedures Involved in the RBFPD

**Figure 5:**
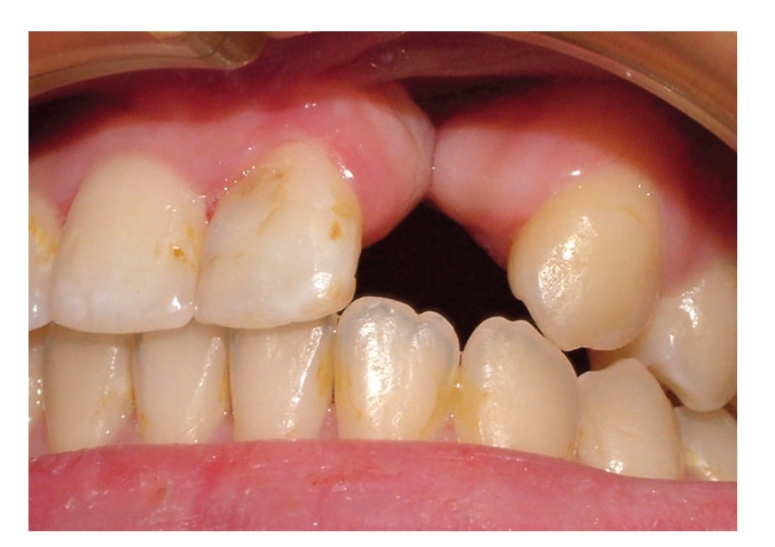
Case 2 with cleft lip and palate.

**Figure 6:**
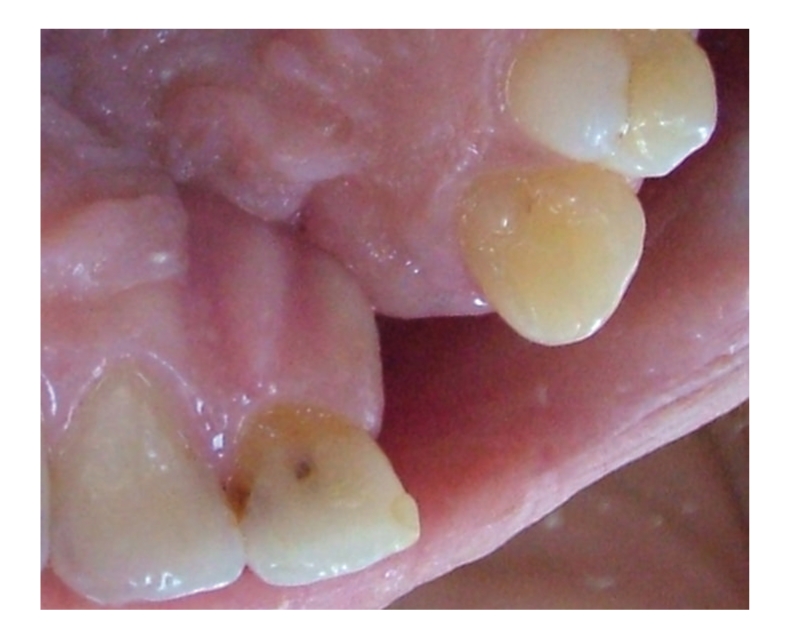
Proximal cavity preparations for the inlays.

First, proximal cavities were prepared for the inlays that would facilitate a well-aligned path of insertion (Figures 5 and 6). All of the internal angles were rounded to facilitate fitting and to reduce the stress concentration. The occlusal portion of the cavity preparation should allow sufficient space to place the polyethylene fiber and composite to ensure a good esthetic result and adequate intracoronal resistance. This was achieved by preparing the isthmus to a width of 1.5 to 2.0 mm at the premolars and 2.5 to 3.0 mm at the molars, while reducing the occlusal surface to a minimum depth of 2.0 to 2.5 mm. The proximal boxes extended gingivally to improve the stability of the restoration, leaving the cervicoproximal cavity margin located in the supragingival enamel. To optimize acid etching, the proximal boxes should have cavosurface angles of 60 to 80 degrees.

**Figure 7:**
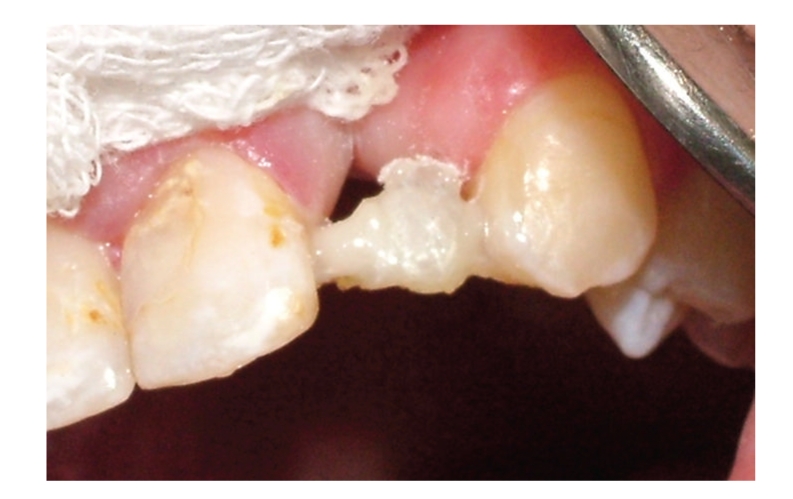
Fiber-reinforced composite.

After cavity preparation, a piece of reinforcing fiber, which had been coated with bonding agent, was packed into the inlay cavity of one abutment tooth and the free ends of the fiber were extended to the inlay cavity of the other abutment tooth (Figure 7).

**Figure 8:**
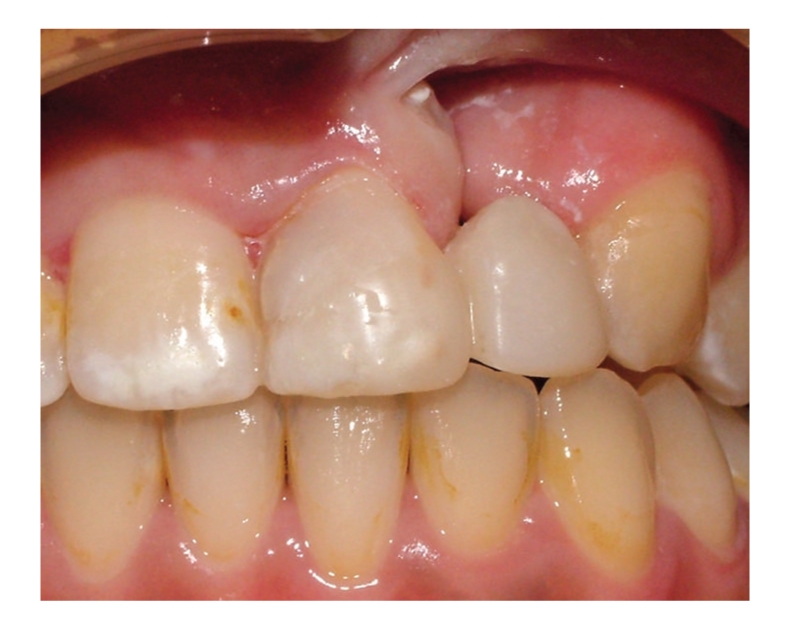
Final restoration.

**Figure 9:**
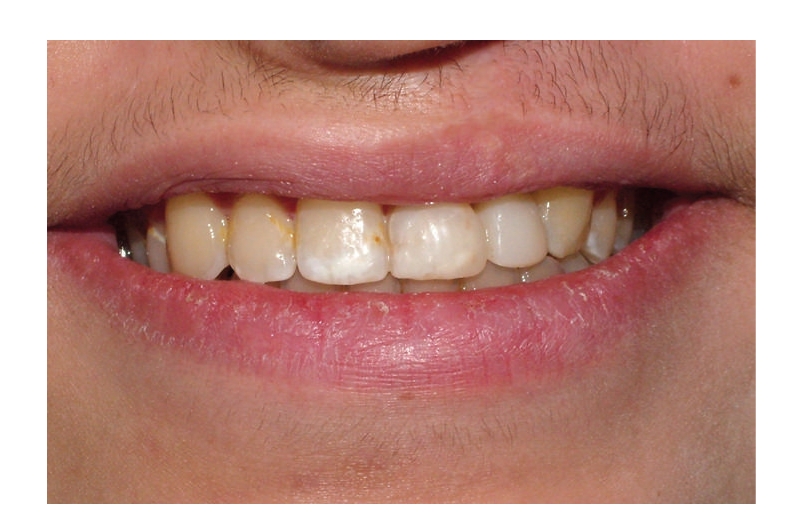
Esthetic view of the final restoration.

The bulk of the crown of the pontic and the inlay cavity restoration of the abutment teeth were formed using a layer of stronger hybrid resin (Clearfil AP-X, Kuraray). The resin restoration was cured for at least 2 minutes with a resin composite-curing unit. Then, the restoration was given a final shaping and polishing (Figures 8 and 9).

## 3. Discussion

Cleft lip and cleft palate are among the most common congenital anomalies. The reported incidence of cleft lip and palate is 2 per 1000 live births in Japan and from 1.25 to 1.43 per 1000 in the United States [7, 8]. When medical and dental interventions improve the appearance and function of a patient with congenital and craniofacial defects, this can have a profound effect on the individual's happiness and productivity. Implant-supported fixed and removable prostheses, overdentures, and traditional fixed and removable prostheses can provide more normal facial contours, an improved smile line, improved arch relationships, and improved function for teens and young adults with facial defects. Implant-supported prostheses can enhance stability, retention, function, and bone preservation. The authors have observed that patients with congenital craniofacial defects often feel more positive about themselves after prosthetic treatment. Patients embarrassed by their teeth and facial appearance are frequently less motivated to maintain good oral hygiene or seek regular dental care, resulting in increased tooth loss and destruction of oral tissues; this exacerbates an existing problem. Early intervention can be extremely beneficial for the patient's well-being [1].

Maxillofacial prosthetic treatment, a combination of fixed, implant-supported, and removable prostheses in conjunction with other dental and medical treatment, may be necessary to obtain the maximum ideal outcome for the patient.

The use of a fixed partial denture may create a number of problems such as the removal of sound tooth structure and difficulty in oral hygiene with reduced gingival and periodontal health. It has been recommended that two abutment teeth be used on each side of the cleft [9].

Well-planned prosthetic, periodontal, and surgical therapy may result in satisfactory function and esthetics, alleviating the deformities. With education and appropriate recall, the patients should be able to maintain their oral health.

When replacing a tooth, the following solutions may be considered: (1) an implant-supported single crown; (2) a conventional fixed partial denture (FPD); and (3) a resin-bonded fixed partial denture (RBFPD). Removable partial dentures should ameliorate the health of the remaining dentition and surrounding oral tissue [10]. With carefully planned prosthetic treatment and adequate checking of oral and denture hygiene, there will be little or no damage to the remaining teeth and periodontal tissues [11]. The type of retainer used influences the survival rate of the dentures [12]. RPDs retained with a telescopic attachment, the so-called rigid design, improve oral function and ensure predictability [13].

A removable dental prosthesis may be used as a provisional form of tooth replacement. Although it can provide good esthetics, a portion of the prosthesis must rest on the soft tissues of the palate and may cause irritation. The removable nature of the prosthesis is a common patient objection. It is used only as a definitive means of tooth replacement when multiple teeth are missing and the edentulous space is too extensive to be spanned by a fixed restoration [5, 7]. For patients with insufficient tissue, it is also used when the traditional hygienic pontic form of a fixed prosthesis does not affect speech production [14].

For cases in which the abutment teeth require no restoration, a resin-bonded fixed dental prosthesis can be used [15, 16]. This conservative option is chosen because it preserves tooth structure. Resin composite restorations have excellent physical properties, marginal integrity, and esthetics. 

On completion of the prosthesis, routine maintenance was performed during two or three patient recalls over the next year. Probing depths varied between 1 and 1.5 mm, and there was no gingival recession or inflammation in the region of the prosthesis. The patients were satisfied and reported no functional or esthetic problems.
